# Accelerating Gas Adsorption on 3D Percolating Carbon Nanotubes

**DOI:** 10.1038/srep21313

**Published:** 2016-02-18

**Authors:** Hui Li, Chenyu Wen, Youwei Zhang, Dongping Wu, Shi-Li Zhang, Zhi-Jun Qiu

**Affiliations:** 1 State Key Laboratory of ASIC and System, School of Information Science and Technology, Fudan University, Shanghai 200433, China; 2 Solid-State Electronics, The Ångström Laboratory, Uppsala University, Uppsala Box 534, SE-751 21, Sweden

## Abstract

In the field of electronic gas sensing, low-dimensional semiconductors such as single-walled carbon nanotubes (SWCNTs) can offer high detection sensitivity owing to their unprecedentedly large surface-to-volume ratio. The sensitivity and responsivity can further improve by increasing their areal density. Here, an accelerated gas adsorption is demonstrated by exploiting volumetric effects via dispersion of SWCNTs into a percolating three-dimensional (3D) network in a semiconducting polymer. The resultant semiconducting composite film is evaluated as a sensing membrane in field effect transistor (FET) sensors. In order to attain reproducible characteristics of the FET sensors, a pulsed-gate-bias measurement technique is adopted to eliminate current hysteresis and drift of sensing baseline. The rate of gas adsorption follows the Langmuir-type isotherm as a function of gas concentration and scales with film thickness. This rate is up to 5 times higher in the composite than only with an SWCNT network in the transistor channel, which in turn results in a 7-fold shorter time constant of adsorption with the composite. The description of gas adsorption developed in the present work is generic for all semiconductors and the demonstrated composite with 3D percolating SWCNTs dispersed in functional polymer represents a promising new type of material for advanced gas sensors.

Nano-structured carbon materials, notably one-dimensional (1D) carbon nanotubes and two-dimensional (2D) graphene, have been explored for their potential in chemical and biological detection applications^1^,^2^,^3^. Although monolayer graphene, via Hall effects, has been reported to be able to detect single molecules^4^, its intrinsic gapless characteristic, and therefrom small current ratio of “on”-state to “off”-state current, inclines to defeat its detection sensitivity in the field-effect transistor (FET) configuration^5^. In contrast, semiconducting single-walled carbon nanotubes (SWCNTs) display orders of magnitude change in conductance upon electrostatic gating or doping^6^. This characteristic makes them promising as active elements in the FET design for sensing. In early studies, FETs each consisting of a single SWCNT in the channel were used as the model system for the study of adsorption processes and properties^7^,^8^. Compared to two-terminal sensing devices, the three-terminal FET sensors are advantageous in yielding multiple electrical responses upon exposure to a target gas. Such responses can be quantified and their likely correlations found based on the well-established device physics, *e.g.* shift in threshold voltage (*V*_t_), variations in drain current (*I*_d_), mobility, *etc*. The advantage with multiple inputs can lend us the opportunity to unveil the mechanism(s) in gas adsorption and desorption.

Despite great potentials, the realization of practical sensors based on individual SWCNTs awaits breakthroughs in material synthesis, fabrication process, and device reproducibility. An alternative is to use SWCNT thin films where the electrical conduction occurs via the networked SWCNTs so as to alleviate fluctuations and uncertainties caused by the aforementioned technological difficulties^9^. An important added advantage is that sensors made of SWCNT thin films can be processed with conventional microfabrication techniques^10^. Field-effect gas sensors with randomly networked SWCNTs have shown the sub-ppm detection capability towards small gas molecules such as NO_2_ and NH_3_ at room temperature^11^,^12^. Hence, this type of sensors remains highly attractive for low-cost and disposable applications as opposed to using individual SWCNTs or monolayer graphene. Since sensitivity and responsivity can improve by incorporating and exposing more nanotubes to gas molecules, it is desirable to stack two-dimensional (2D) SWCNT networks to a three-dimensional (3D) architecture. This stacking can increase the SWCNT areal density. However, SWCNT films obtained by means of facile solution-processing often consist of nanotubes with a sub-monolayer surface coverage and conduct current via a 2D percolation network^13^. A 3D structure cannot establish due to a so-called self-limiting mechanism where the first-adsorbed SWCNTs tend to block further nanotube adsorption onto the substrate^14^. A general strategy is, therefore, to form a 3D high-specific-surface mesoporous composite by dispersing the SWCNTs in a functional polymer matrix of high gas permeability^15^. This approach is particularly appealing since the composite still retains the attractive mechanical properties as well as the processing advantages of the polymer. It has, indeed, been found that the SWCNT/polymer composite displays an improved sensitivity in comparison to using SWCNTs alone^16^,^17^. There have been numerous reports on the development of gas sensors based on various SWCNT/polymer composites processed in solution, with a primary focus on studying the equilibrium adsorption of gas molecules on the composites. Their adsorption isotherms and kinetics are, however, less investigated and remain poorly understood.

Here, we have fabricated high-fidelity FET sensors with a well-characterized composite-semiconductor for a systematic gas adsorption study^18^. We will establish that assembling nano-sized sensing components into a 3D sensing layer presents an advantageous and practical approach. The composite comprises the air-stable semiconducting π-conjugated poly(9,9-dioctylfluorene-co-bithiophene) (F8T2)^19^ and highly purified semiconducting SWCNTs. These two materials share a similar aromatic structure and tend to hybridize due to their strong π-π interaction^18^,^20^. With the SWCNT density exceeding the percolation threshold, there is a high likelihood that the nanotubes will intersect with one another and form continuous electrical paths in the polymer, *i.e.* a 3D percolating conductive network^21^.

## Results

**Figure 1:**
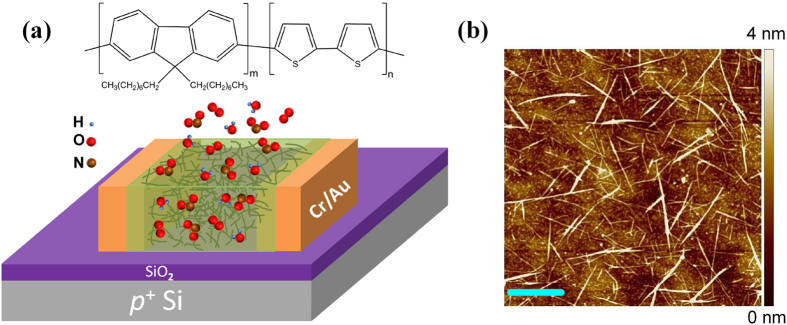
Basic device structure. (**a**) Schematic structure of a back-gate FET with an F8T2/SWCNT composite film as the semiconductor channel. Random adsorption of gas molecules in the channel film is schematically illustrated. Inset: the chemical structure of F8T2. (**b**) AFM image in height mode of the composite channel. Scale bar, 1 μm.

A schematic representation of our SWCNT/F8T2-composite FET is depicted in Fig. 1a, with the Si substrate and SiO_2_ layer as the gate electrode and gate dielectric, respectively. The device fabrication is detailed in Methods. The semiconductor channel between the two Au electrodes (*i.e.* the source and drain terminals) is typically 20 μm long and 200 μm wide. A high density of nanotubes is well dispersed in the polymer as shown in the micro-image of Fig. 1b obtained by means of atomic force microscopy (AFM). For sensor evaluation, NO_2_, one of the most concerned air pollutants, is used as a test gas.

In characterizing carbon-based (SWCNT and graphene) FET sensors in the ambient environment, caution must be exercised since a large hysteresis in *I*_d_ can appear when the gate voltage (*V*_g_) is swept forwardly and then reversely^22^,^23^. The hysteresis in *I*_d_ represents an instability issue of the sensor device and can render the sensing outcome invalid. This is especially true for detecting gas molecules at low concentrations where weak signals can be marred by false variations due to device instability. It is well known that the adsorption of H_2_O/O_2_ molecules from the ambient environment induces hysteretic behaviors and irreproducible transport phenomena in the SWCNTs and their FETs^24^,^25^. A simple N_2_ purging commonly used in gas sensing experiments is unable to completely remove the adsorbed H_2_O/O_2_ molecules at room temperature. In order to reduce the cumulative hysteretic effects, a treatment in vacuum at high temperature for long duration is required^22^. But such a demanding treatment can degrade the thermal stability of the polymer, and thereby complicating the sensing pursuit. Besides, gas sensing is usually practiced in non-controlled environments as opposed to experimenting in the laboratories.

**Figure 2:**
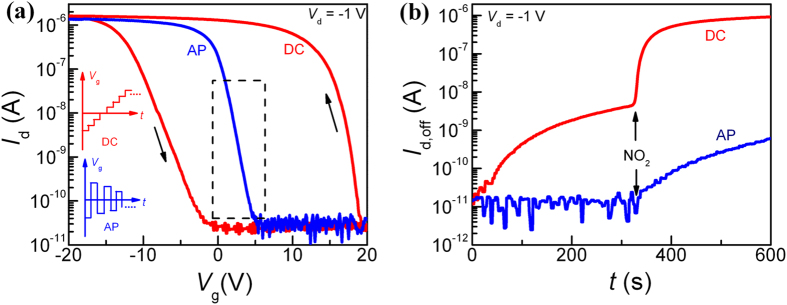
Electrical characteristics and gas-sensing behavior. (**a**) Transfer characteristics of the 13-nm-thick composite-FET with *V*_g_ sweeping in DC or AP mode, with the arrows indicating the sweep direction. Insets: schematic of the DC and AP modes of *V*_g_ sweep. Dashed rectangle: effective detection window, EDW. (**b**) Electrical response of the composite sensor to 50 ppm NO_2_ in DC or AP mode, with the arrow indicating the time point for the sensor to be exposed to NO_2_. In the DC mode *V*_g_ is fixed at 20 V, while in the AP mode the train of the *V*_g_ pulses is +20 V/−20 V.

The typical *p*-type transfer characteristics, *i.e. I*_d_ versus *V*_g_, of an SWCNT/F8T2-composite FET operated in dry N_2_ are depicted in Fig. 2a, at a constant drain bias *V*_d_ = −1 V. A severe hysteresis in *I*_d_ is evident with two distinct *V*_t_ values for the *I*_d_-*V*_g_ curves when *V*_g_ was first continuously swept from −20 to 20 V and then back to −20 V as depicted by one of the insets in Fig. 2a, *i.e.* in DC mode. The *V*_t_ uncertainty has severe consequences; when the device was biased at *V*_g_ = 20 V and *V*_d_ = −1 V, a substantial increase in the “off”-state current, *I*_d,off_, of the device by 3 orders of magnitude over about 300 s is found in Fig. 2b. This increase in *I*_d,off_ actually slows down significantly beyond 200 s and the slowly-varying current level would conventionally be used as the baseline for sensing. When exposing the FET to NO_2_, *I*_d,off_ drastically increases immediately. However, we will show that such a wished-for response does not result from NO_2_ alone and it arises from a collaborative effort of both the adsorbed NO_2_ molecules and the interfering H_2_O/O_2_ molecules.

In order to facilitate a quantitative study of the SWCNT/F8T2-composite FETs as a sensor device, a unique electrical characterization procedure with *V*_g_ pulses of alternating polarities, *i.e.* AP mode, is employed to eliminate the *I*_d_ hysteresis^25^,^26^,^27^. In the AP mode, each positive pulse is followed by a negative pulse of equal amplitude, as illustrated by the other inset of Fig. 2a. The immediate application of a negative *V*_g_ pulse aims at depriving away the cumulated charges during the positive *V*_g_ pulse; the reverse by exchanging the polarity or order of *V*_g_ pulses is also operational. This practice with the AP mode has indeed resulted in stable electrical characteristics with hysteresis-free transfer curves in Fig. 2a (i.e. the forward and reverse curves completely overlap) and a horizontal baseline in Fig. 2b. The pulsed measurement allows, as expected, the current to be monitored without interference from the ambient environment. Upon exposure to NO_2_, a much weaker response than that in the DC mode is obtained in Fig. 2b. This comparison indicates that the environmental interference can complicate the sensing process and make the sensing results unpredictable. Apparently, the use of the AP mode effectively suppresses the charge transfer between the adsorbed H_2_O/O_2_ molecules and the SWCNTs without affecting that between the adsorbed NO_2_ molecules and the SWCNTs. However, the NO_2_ adsorption on nanotubes represents a complex process, which involves possible charge-transfer-mediated chemical reactions^28^,^29^. These reactions are hardly affected by pulsing *V*_g_ as used in this study. Therefore, unless otherwise stated, all our gas-sensing measurements in the remainder of this work are performed in the AP mode. It is worth reminding that the competition for adsorption sites by H_2_O, O_2_, and NO_2_ prevails even if the hysteresis originating from the adsorbed H_2_O/O_2_ molecules is suppressed by operating the sensor device in the AP mode. Since the density of the active adsorption sites in the composite is invariant, no further adsorption can take place once a specific site is occupied. A lower response in air than in N_2_ at the same NO_2_ concentration observed in Supplementary Fig. S1 indeed confirms that the adsorption sites already occupied by O_2_/H_2_O molecules are not accessible to NO_2_. This observation motivates the adsorption kinetics study (below) of NO_2_ in a 3D SWCNT percolating network dispersed in the F8T2 matrix, all in N_2_ atmosphere.

**Figure 3:**
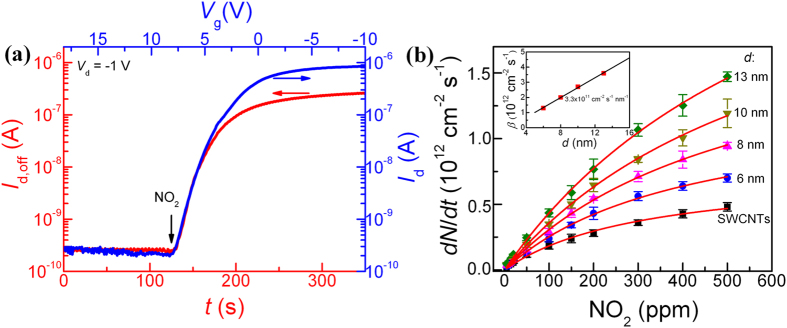
Extraction of adsorption rate. (**a**) Overlap in the subthreshold regime of the transfer characteristics *I*_d_-*V*_g_ and the time-response characteristics *I*_d,off_-*t* of the 13-nm-thick composite-FET to 50 ppm NO_2_ exposure. The train of the *V*_g_ pulses for the *I*_d,off_-*t* curve is +8 V/−8 V. (**b**) Dependence of 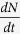 on NO_2_ concentration for a series of composite films of different thickness, *d*, along with an SWCNT-network film. Solid lines: fitting results according to equation (2). The data points and error bars represent the average value and the standard deviation, respectively, from six separate sensors of the same material. Inset: dependence of *β* on *d*, solid line is the linear curve fitting with a slope of 3.3×10^11 ^cm^−2 ^s^−1 ^per nm.

The majority of recent reports^30^,^31^,^32^,^33^, including theoretical studies, conclude that the charge transfer between the adsorbed gas molecules and SWCNTs is the primary sensing mechanism. The charge transfer can lead to the so-called molecular gating effect, *i.e.* the accumulated charge causes *V*_t_ to shift and the conductance to change, and can eventually turn the transistor state from “off” to “on” or the other way around. However, the role of gas adsorption in modulating the Schottky barrier at the SWCNT/metal contact as well as the consequences in gas sensing remains to be clarified^34^,^35^,^36^. In an SWCNT network, a further sensing mechanism at work is the modulation of the energy barrier at the tube-tube junctions by adsorbed molecules^37^,^38^,^39^. Upon NO_2_ exposure, *I*_d,off_ initially follows an exponential increase with time, *t*, and then tends to saturate for longer time, see Fig. 3a. This *I*_d,off_-*t* behavior is induced by the molecular gating effect, and it resembles very well the transfer *I*_d_-*V*_g_ characteristics. These two curves are plotted together in Fig. 3a with their subthreshold regimes overlapping. This practice leads to a correlation between the electrostatic and molecular gating that can be exploited to quantify the adsorption rate of NO_2_, 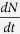, with *N* as the areal density of occupied adsorption sites or adsorbed molecules. From the slope of the time-response curve and that of the transfer characteristic curve in the semi-logarithmic scale, *i.e.* 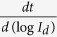 and 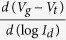, respectively, the rate of *V*_t_ change during adsorption, 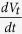, can be obtained for a constant *V*_g_. Of the first-order approximation, which assumes that the gas molecules are uniformly adsorbed in the composite channel, the following is readily obtained,


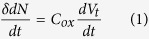


where, *C*_ox_ is the capacitance of the gate dielectric per unit area and *δ* the amount of transferred charge per molecule. Both experimental work and theoretical calculations have shown that the electron transfer from SWCNTs to NO_2_ is approximately 0.1 electrons per NO_2_ molecule^30^,^40^,^41^. With 100-nm-thick SiO_2_, 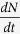 for the SWCNT/F8T2-composite sensors and the SWCNT-network-only reference is plotted as a function of NO_2_ concentration in Fig. 3b. In both cases with composite and SWCNT-network, 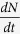
increases with increasing NO_2_ concentration and the rate is higher for the composite than for the SWCNT-network. Moreover, the gas adsorption in the composite sensors is dependent on the thickness of the composite films. In our work, the thickness of the composite films is controlled by varying the dip-coating time. Longer dip-coating times yield thicker composite films and the resulting sensors with larger 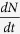, simply due to more SWCNTs in the films.

The dependence of 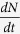 on NO_2_ concentration is found to follow the Langmuir adsorption isotherm equation^42^:


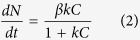


by assuming a fixed density of independent absorption sites for the gas molecules to bind and no interaction between the molecules. Here, *C* is the NO_2_ concentration and *k* the Langmuir constant, while *β* represents 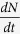
at saturation. The excellent fit of the experimental data by equation (2) in Fig. 3b yields *β* = 0.8, 1.3, 2.0, 2.7, and 3.6×10^12 ^cm^−2 ^s^−1^ for the SWCNT-network and composite films of 6, 8, 10, and 13 nm thickness, respectively. It is remarkable that 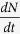 of the 13-nm thick composite film is almost 5 times that of the SWCNT-network. It is important to observe a linear relationship between *β* and the thickness of the composite films, *d* (inset in Fig. 3b), with a slope of 3.3×10^11 ^cm^−2 ^s^−1 ^per 1 nm. Higher SWCNT areal densities in thicker composite films are favorable in increasing the adsorption rate and thereby the gas sensitivity.

**Figure 4:**
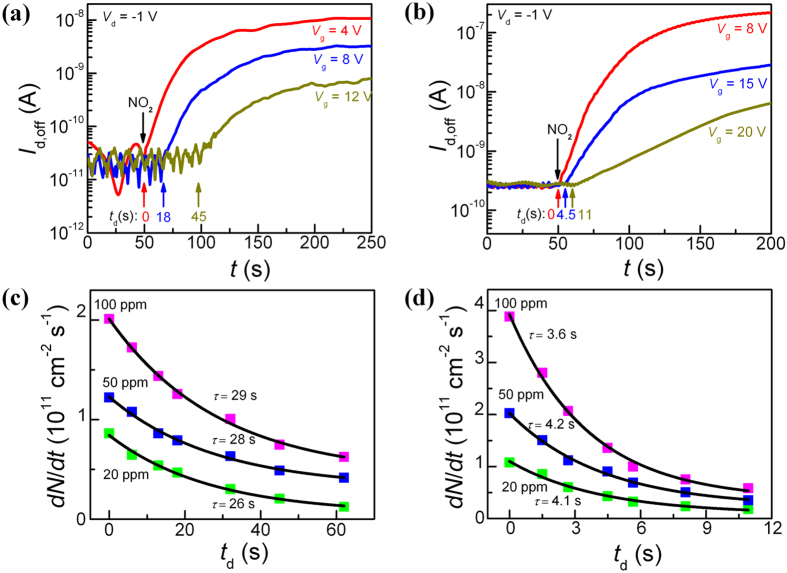
Adsorption kinetics in SWCNT-network and SWCNT/F8T2-composite. (**a,b**) Gas response of the SWCNT-network and the 13-nm-thick SWCNT/F8T2-composite, respectively, with different *V*_g_ pulses. Only positive *V*_g_ pulses are shown here. Black arrows: time point for the sensor to be exposed to NO_2_. Colored arrows: the onset time points at which *I*_d,off_ starts to increase. Delay in time from gas feeding to sensor response strongly dependent on *V*_g_. (**c,d**) Dependence of 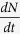 on *t*_d_ for the SWCNT-network and SWCNT/F8T2-composite, respectively. Solid lines: fits results according to the first derivative of equation (3).

Operating a sensor device in its subthreshold regime is generally considered advantageous due to an exponential dependence of *I*_d_ on *V*_t_ that can shift by gas adsorption^43^. Hence, this regime presents an effective detection window, EDW, with high sensitivity, marked by the rectangle in Fig. 2a. When the device is biased in its “off” state on the far right of EDW, *i.e.* at large positive *V*_g_, *I*_d,off_ stays low close to the noise level even if gas molecules have been adsorbed. This “off” state can change when the adsorbed charge is accumulated to a level sufficient to pull the device into EDW. In practice, there will be a time delay from gas feeding to sensor response. In the AP mode, a train of *V*_g_ pulses of alternating polarity with identical amplitude, *e.g.* +20 V/−20 V, is used to characterize gas sensing. When a negative *V*_g_ pulse is applied, the device is in its “on” state while a positive *V*_g_ pulse sets the device to its “off” state. Hence, the device is consecutively switched between the “on” and “off” states during its exposure to the test gas NO_2_. The total gas exposure time used in this study is 200 s. As illustrated in Supplementary Fig. S2, the device in its “on” state immediately responds to the gas feeding, while it in its “off” state exhibits a certain time delay for both SWCNT-network and composite. This delay depends on the value of the positive *V*_g_ pulse relative to EDW and is confirmed in Fig. 4a,b: the onset of the substantial increase in *I*_d,off_ is pushed to longer times for larger positive *V*_g_ pulses. Concurrently, *I*_d,off_ tends to saturate at lower current levels because the adsorption sites occupied by NO_2_ during the delay period do not contribute to the sensor response. At the extreme, the sensor would not respond at all if *V*_g_ could assume an extremely large positive value without device breakdown. Hence, a delay time for gas response, *t*_d_, exists between the “on” and “off” states. During this time, *I*_d,off_ is unaffected by the adsorbed gas molecules, but beyond *t*_d_, it starts to respond. This feature is important as it can help understand the adsorption kinetics. Furthermore, *t*_d_ can be accurately controlled by varying the *V*_g_ pulses. The dependence of 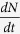 on *t*_d_ for the SWCNT-network and composite is summarized in Fig. 4c,d, respectively. Increasing *t*_d_ leads to an exponential decay of 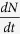 due to the reduction of the available adsorption sites, in accordance to the Langmuir theory in which *N* at any time can be described as^44^:


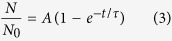


Here, *N*_0_ is the total areal density of the available adsorption sites, *A* the amplitude of the response, and *τ* the time constant. The solid lines in Fig. 4c,d represent the fitting results of the experimental data points by the first derivative of equation (3). The derived *τ* is found independent of gas concentration, as expected, and it is about 4 s for the composite and 28 s for the SWCNT-network (*i.e.* 7 times larger). It is, therefore, understood that the composite is characterized by a more rapid response and higher sensitivity than the SWCNT-network does in a field-effect sensing device. The demonstrated high gas-sensing performance with the SWCNT/F8T2-composite FET sensor is primarily due to the formation of a 3D SWCNT percolating network in the F8T2 matrix. However, other important roles of the polymer should not be overlooked since it can assist absorbing, concentrating, and retaining the gas molecules around the dispersed SWCNTs^45^,^46^. As a result, the interaction between the adsorbed gas molecules and nanotubes can be enhanced, apart from the polymer acting as the matrix for a uniform distribution of SWCNTs.

## Discussion

Field-effect gas sensors with their sensing membrane placed at the surface are expected to generate the most sensitive outcome. Operating such sensors in the ambient environment is, however, susceptible to interferences from the inevitable adsorption of H_2_O/O_2_ molecules on the sensing membrane. The interaction in the form of charge transfer between the sensing membrane and the H_2_O/O_2_ molecules has been found to cause a severe current hysteresis; hysteresis generally represents a specific type of device instability. The instability can be the cause responsible for a false interpretation of sensing experiments. In this work, we have shown that this hysteresis, and thereof drift of the sensing baseline, in the SWCNT/F8T2-composite based field-effect sensor can be effectively suppressed by operating the device in the AP mode. This method allows for the establishment of an analytical procedure for quantification of the gas adsorption via the unique subthreshold-regime detection window of the FET sensors. Without the influence of hysteresis, we have further demonstrated that the adsorption rate, and thereby the detection sensitivity, can be significantly enhanced by exploiting volumetric effects via the formation of a 3D percolating SWCNT network in the polymer. The function of the gas-molecule-permeable polymer is multiple and should not be overlooked. This system with the SWCNT/F8T2-composite has allowed us to explore the adsorption isotherms and the kinetics of gas adsorption in detail, which is of great significance because, in addition to gas-detection applications, the adsorption process plays an important role in many fields of surface science ranging from material sciences to biochemistry and nanotechnology.

## Methods

### Solution processing and sensor fabrication

The F8T2 (Sigma-Aldrich) used as purchased was dissolved in toluene (Sigma-Aldrich) in a sonic bath. Highly purified 98% semiconducting SWCNTs (NanoIntegris) were then ultrasonically added into the F8T2/toluene solution (0.1 mg mL^−1^) followed by centrifugation at 13300 g for 60 min. The supernatant (15 mL) was carefully decanted for the sensor fabrication. Highly doped *p*-type Si wafers with a 100-nm thermally grown SiO_2_ layer were used as the substrate. The SiO_2_/Si substrate was pre-cleaned by, in this order, sonication in acetone, isopropyl alcohol, and deionized water. Conventional electron-beam evaporation in combination with lift-off process was used to make an electrode pattern with a Ti/Au bilayer of 5 nm/50 nm thickness. The channel area was defined using a second photoresist layer. For each experiment, one chip was sliced out of the wafer and a thin film of the F8T2/SWCNT composite was deposited by dip-coating the chip in the polymer solution. The thickness of the composite films is controlled by varying the dip-coating time. The chip was then carefully pulled out and baked at 90 °C to remove the residual solvent. The film formed outside the channel region was stripped off in acetone along with the photoresist. After blown-dry in N_2_, a 10-min hot-plate baking at 90 °C completed the device fabrication. The film thickness was measured using AFM (Dimension 3100 with Nanoscope IIIa controller, Veeco) operated in tapping mode under ambient conditions. For 2, 12, 24, and 48 h of dip-coating, composite films of 6, 8, 10, and 13 nm average thickness, respectively, were obtained. All the processes were performed under ambient conditions. For reference SWCNT-network FETs, the chip fabrication was identical to the process described above and the SWCNT conduction channel was formed by dip-coating the chip in the SWCNT/surfactant solution.

### Gas sensing measurements

The gas sensing experiment was performed in a homemade vacuum-sealed stainless steel chamber equipped with a Keithley 4200 semiconductor parameter analyzer in a controlled atmosphere. The desired NO_2_ concentrations were attained by intermixing calibrated commercial NO_2_ with purified N_2_ at specific ratios using respective mass flow controllers (SLA5850E, Brooks). When exposed to different concentrations of NO_2_, the response was measured by monitoring the change in the current-voltage (*I*-*V*) characteristics of the sensors. In the AP mode, the *V*_g_ pulse width is set to be 0.5 s. All sensing experiment was carried out at room temperature.

## Additional Information

**How to cite this article**: Li, H. *et al.* Accelerating Gas Adsorption on 3D Percolating Carbon Nanotubes. *Sci. Rep.* **6**, 21313; doi: 10.1038/srep21313 (2016).

## Supplementary Material

Supplementary Information
