# Supplementary material for: Accelerating Gas Adsorption on 3D Percolating Carbon Nanotubes
Source: Sci Rep. 2016 Feb 18;6:21313. doi: 10.1038/srep21313 (PMC4758076; doi:10.1038/srep21313)
Supplement: Supplementary Information [file srep21313-s1.pdf]

Accelerating Gas Adsorption on 3D Percolating Carbon Nanotubes

Hui Li, Chenyu Wen, Youwei Zhang, Dongping Wu, Shi-Li Zhang, and Zhi-Jun Qiu

Supplementary Information:

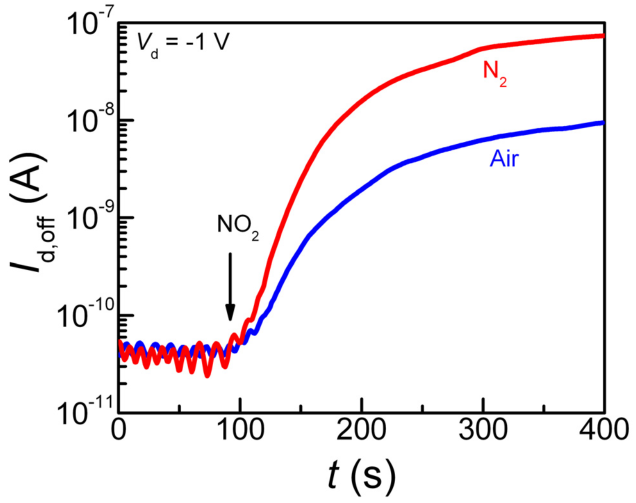

Figure S1. Gas-sensing behavior in different atmospheres. Electrical response, $I_{d,off}$, of the 13-nm-thick composite-FET sensor to 20 ppm NO_2 in air or N_2. The same train of V_g pulses is applied with +8 V/-8 V.

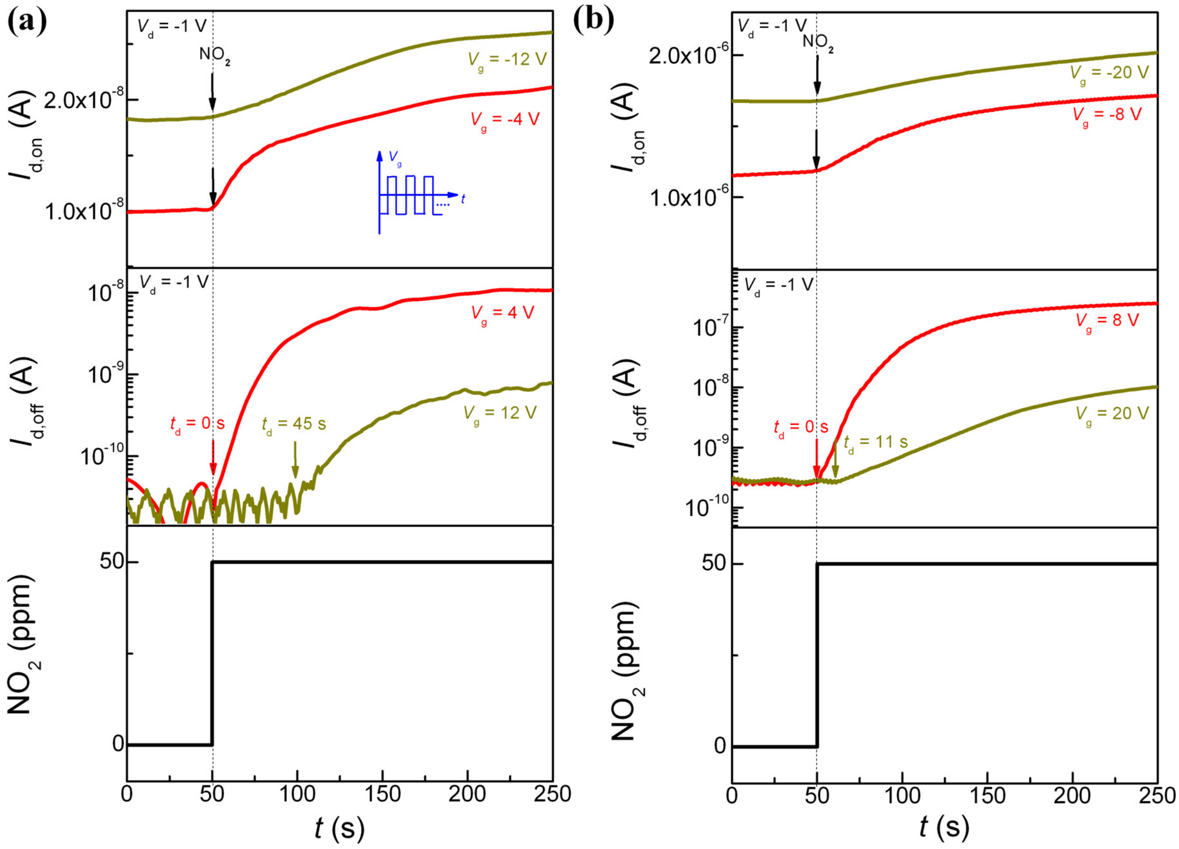

Figure S2. Time delay of gas response. (**a,b**) Gas response of the SWCNT-network and the 13-nm-thick SWCNT/F8T2-composite at different V_g pulses (schematically shown in inset), respectively. Top and middle panels show the variation of the "on"-state current, $I_{d,on}$, and $I_{d,off}$, corresponding to negative and positive V_g pulses, respectively, upon exposure to 50 ppm NO_2 (bottom panel). Black arrows: time point for the sensor to be exposed to NO_2. Colored arrows: the onset time points at which $I_{d,off}$ starts to increase. There is a time delay, t_d, in the gas response between $I_{d,on}$ and $I_{d,off}$, depending on the amplitude of V_g pulses. t_d = 0 s is observed for the SWCNT-network and composite at V_g pulses of +4 V/-4 V and +8 V/-8 V. V_g pulses of +12 V/-12 V and +20 V/-20 V induce t_d = 45 s and 11 s for the SWCNT-network and composite, respectively.
